# A multiethnic association analysis of hyperuricaemia with cardiovascular risk in rural and urban areas in Chinese adults

**DOI:** 10.1038/s41598-021-02740-y

**Published:** 2021-12-03

**Authors:** Leilei Liu, Juan Lei, Linyuan Zhang, Nana Ma, Zixuan Xu, Lian Peng, Chan Nie, Jianqin Zhong, Xiao Zhang, Feng Hong

**Affiliations:** 1 School of Public Health, The Key Laboratory of Environmental Pollution Monitoring and Disease Control, Ministry of Education, Guizhou Medical University, Guiyang, 550025 China; 2 Guiyang Center for Diseases Control and Prevention, Guiyang, 550003 China; 3 Center for Diseases Control and Prevention of Yunyan District, Guiyang, 550004 China

**Keywords:** Cardiovascular diseases, Risk factors, Endocrinology, Epidemiology

## Abstract

Comprehensive research on rural–urban disparities in the association of hyperuricaemia (HUA) with cardiovascular disease (CVD) in China, especially among minority groups, is limited. We explored the HUA-CVD relationship between rural and urban areas within ethnic Chinese groups. We included Dong, Miao, and Bouyei adults in Southwest China from the China Multi-Ethnic Cohort Study. Multivariable logistic regression models were used to assess the relationship between HUA and CVD in both residences. We performed stratified analyses by sex and age. The study population included 16,618 people (37.48% Dong, 30.00% Miao, and 32.52% Bouyei) without a reduced estimated glomerular filtration rate. We identified 476 (188 Dong, 119 Miao, and 169 Bouyei) and 175 (62 Dong, 77 Miao, and 36 Bouyei) CVD cases in rural and urban areas. Compared to urban residents, an at least 49% increased CVD risk (adjusted OR 1.49, 95%CI 1.06–2.08 for the Dong ethnic group; 1.55, 1.07–2.25 for the Bouyei ethnic group) and a 1.65-fold elevated coronary heart disease risk (1.65, 1.03–2.64) related to HUA was present in rural residents. Moreover, HUA was positively associated with increased risk of CVD and coronary heart disease in rural women (2.05, 1.26–3.31; 2.11, 1.19–3.75) and rural older adults (1.83, 1.22–2.75; 2.32, 1.39–3.87) among the Bouyei ethnic group, respectively. We found rural elderly individuals with HUA among the Dong ethnic group had a 52% elevated risk of CVD (1.52, 1.05–2.21); furthermore, an at least 79% increased risk of stroke related to HUA was observed in women (2.24, 1.09–4.62) and elderly people (1.79, 1.02–3.13) in rural areas among the Dong ethnic group. But a positive association was not found among the Miao ethnic group. Screening early-onset HUA patients may be helpful for the control and prevention of CVD in rural residents, especially for women and older adults living in a rural community, among the Dong and Bouyei ethnic groups in China.

## Introduction

Despite progress in declining cardiovascular disease (CVD) mortality worldwide, CVD remain the leading cause of death globally, and more than half of CVD deaths occur in Asia^1^. Moreover, the reduction in CVD burden was under 25% over the past 30 years in Southwest China^2^. Racial and ethnic disparities in CVD burden persist. There is a need to evaluate associated risk factors in light of the severe disease burden.

Several known risk factors, such as hypertension, diabetes mellitus, and hyperlipidaemia, have been verified to be related to CVD burden. The attributable CVD risk associated with hyperuricaemia (HUA) remains unclear. Although there is epidemiological evidence about the relationship between HUA and the risk of CVD^3–7^, less is known about the potential rural–urban differences in the relationship^8^. Previous studies have focused only on rural areas^9^ or urban residences^10^, or they have combined the data of rural and urban residents^11,12^, the comparison of rural–urban differences in different studies is limited. Furthermore, there are controversial conclusions regarding the relationship between HUA and the risk of CVD among different sexes and ages in rural and urban areas^5,8,13,14^. Additionally, information about the impact of different ethnic groups on the association in different residences was unavailable. Consequently, some questions remain regarding the unexplained rural–urban variation in the HUA-CVD association. We sought to evaluate these questions in the China Multi-Ethnic Cohort (CMEC) Study^15^.

This cross-sectional survey using data collected in the CMEC study^15^ at baseline aimed to elucidate whether the association between HUA and the risk of CVD varies by residence in adults from Dong, Miao, and Bouyei populations living in Southwest China.

## Methods

### Study population

We collected baseline data from the CMEC Study^15^. Detailed descriptions of the study design and study population of the baseline survey have previously been described^15^. In summary, a multistage, stratified cluster sample of 18,790 participants aged 30–79 years old, which included 7,239 Dong, 5,559 Miao, and 5,992 Bouyei people, were recruited from Guizhou Province of Southwest China during July 2018 and August 2019. Participants were excluded because of (1) missing data of serum uric acid (SUA) or serum creatinine (Scr) levels (n = 1,726); (2) fasting time less than eight hours (n = 95); and (3) reduced estimated glomerular filtration rate (eGFR)^16^ < 60 mL/min per 1.73m^2^ (n = 351). We finally selected 16,618 eligible people (6,228 Dong, 4,986 Miao, and 5,404 Bouyei adults).

Ethical approval was granted by the Sichuan University Medical Ethical Review Board (K2016038) and the Research Ethics Committee of The Affiliated Hospital of Guizhou Medical University (2018[094]). Written informed consent was obtained from all participants before taking part in the study. All methods were carried out following relevant guidelines and regulations.

### Covariates assessment

Information on socioeconomic status, medical history, and health behaviours (cigarette smoking, alcohol consumption, and physical activity) were assessed by a questionnaire. The definitions of these abovementioned variables are presented in detail in the Cohort Profile^15^. A current smoker was defined as someone who smoked more than 100 cigarettes during their lifetime^17^. A former smoker was defined as someone who met the above criteria before the half-year and did not smoke during the past half-year, and the others were never smokers^17^. An alcohol drinker was defined as someone who drank at least once per week in the prior year^18^. Total physical activity level was assessed based on job-related physical activity, transportation physical activity, leisure-time physical activity, and housework for each participant and converted to the hours of metabolic equivalent tasks per day (METs h/d)^19^. A family history of CVD referred to the self-reported CVD (coronary heart disease [CHD], stroke, or arrhythmia) from at least one first-degree relative (parent, sibling, or natural child of the participants). The total energy intake (kcal/week) was estimated by summing the number of servings per week of alcohol, tea, oil, and beverages, rice, cooked wheat-based food, vegetables, fruits, meat, etc., which covered the most commonly consumed food groups in Southwest China, by the following manner: first, the unit energy of each type of food was calculated based on, the China food exchange lists, the China Food Composition Tables (2018), and food consumption of Guizhou Province; second, data on the consumption peer week of each type of food were from the food frequency questionnaire; finally, matrix multiplication was conducted based on what was mentioned.

Anthropometric measures, height, and weight, for body mass index (kg/m^2^) calculation, were taken from participants during their encounters, were measured twice, and the means were used for the present analysis. Blood pressure was measured in the upper arm using a calibrated electronic sphygmomanometer (HEM-7136AFuzzy, Omron, Kyoto, Japan) with the participant in a seated and upright position, and they were prohibited to exercise, smoke, and drink alcohol, coffee, or tea, at least 30 min before the measurement. At least three blood pressure measurements were taken after five minutes of rest. We then measured again if the difference between the two measurements was 10 mmHg and used the average value for analysis.

We collected venous blood in the morning after an overnight fast for at least eight hours, one tube of serum, and two tubes of ethylenediaminetetraacetic acid anticoagulant. Additionally, we transported the blood samples under cold chain logistics (-80℃) to Guizhou KingMed Diagnostics Group Co., Ltd.

A fully automatic biochemical analyser (Hitachi 7180, Tokyo, Japan) was used to measure the biochemical indicators, including fasting plasma glucose, triglycerides, total cholesterol, high-density lipoprotein-cholesterol, low-density lipoprotein-cholesterol, Scr, and SUA.

Strict quality control measures were applied for all stages of blood biochemical collection and testing based on national standard operational procedures, such as the training of staff, calibration verification of experiment instruments, and data verification (5% data were randomly selected for re-check and re-test was carried out for abnormal data), to ensure the precision, repeatability, stability, and reliability of laboratory measurement data generation. “The Xiangya equation”^16^, which was applied to a multi-ethnic Chinese population, was used to calculate eGFR, a measure of renal function decline, was derived from data indicating sex, age, and Scr, and reduced eGFR was defined as < 60 mL/min per 1.73 m^2^.

### Definitions of exposure and outcome

In line with many previous studies, we defined HUA as an SUA measurement ≥ 7 mg/dL (420 µmol/L) in men and ≥ 6 mg/dL (360 µmol/L) in women^20^. The endpoint of interest was CVD, and we used data on self-reported information based on a questionnaire. The participants were asked whether they had a reported history of physician-diagnosed CHD, stroke, or arrhythmia from second-level and above hospitals, patients were defined as having one or more of the conditions. The self-reported CVD definition has been verified and used in many population-based studies^21–23^.

### Statistical analyses

The population characteristics were presented using medians with the interquartile range for continuous variables and frequencies with percentages for categorical variables. We compared these participant characteristics between CVD patients and people without CVD. Differences between the two groups were examined using the Mann–Whitney *U* test or chi-square tests for continuous and categorical variables, respectively. We also calculated the prevalence of CVD. The association of HUA with the risk of CVD was analyzed in multivariable logistic regression models to calculate the odds ratio (OR) and 95% confidence interval (CI) adjusted for sex, age, educational attainment, annual household income, tobacco smoking status, alcohol drinking status, total physical activity, total energy intake, family history of CVD, body mass index, and systolic blood pressure, fasting plasma glucose, and triglycerides levels, except for the stratified variables.

All statistical analyses were performed with SAS version 9.1 (SAS Inst. Inc., Cary, NC). All figures were plotted using STATA version 16 (STATA Corp, College Station, TX). Throughout the experiment, we referred to statistical significance as a two-sided *P* value less than 0.05.

## Results

### Basic characteristics of the study population

Participants’ characteristics by CVD status in rural and urban areas among the Dong, Miao, and Bouyei ethnic groups are in Tables 1 and 2. In rural areas, compared with people without CVD, participants with CVD were older, had a lower level of total physical activity, total energy intake, high-density lipoprotein-cholesterol, and eGFR but higher level of systolic blood pressure, diastolic blood pressure, SUA, and Scr among all three ethnic groups; moreover, CVD patients had a higher proportion of men and alcohol consumption weekly, and had a lower level of high-density lipoprotein-cholesterol among the Dong ethnic group, higher level of triglycerides among the Miao ethnic group, and were more frequently men and higher level of body mass index, fasting plasma glucose, triglycerides, and low-density lipoprotein-cholesterol among the Bouyei ethnic group (all *P* < 0.05) (Table 1). In urban areas, people with CVD were older, had a higher level of systolic blood pressure, fasting plasma glucose, and Scr, but had a lower level of eGFR among all three ethnic groups; also, those with CVD had a lower level of total energy intake but had a higher level of body mass index, triglycerides, and SUA among the Dong ethnic group, and had a lower level of education attainment and total physical activity among the Miao and Bouyei ethnic group, and had a higher level of SUA among the Miao ethnic group (all *P* < 0.05) (Table 2).

**Table 1:** Basic characteristics of the Dong, Miao, and Bouyei ethnic groups by cardiovascular disease status in rural areas.

Variables	Dong ethnic group		*P*	Miao ethnic group		*P*	Bouyei ethnic group		*P*
	non-CVD	CVD		non-CVD	CVD		non-CVD	CVD	
No. of participants	4658	188		3431	119		4554	169	
Age (years)	52.40 (45.83–61.13)	62.04 (54.11–68.05)	< 0.001	50.55 (42.67–61.03)	62.52 (53.57–68.57)	< 0.001	50.82 (43.77–57.98)	59.60 (52.82–65.77)	< 0.001
Men, n (%)	1550 (33.28)	84 (44.68)	0.001	1223 (35.65)	46 (38.66)	0.501	1335 (29.31)	68 (40.24)	0.002
**Education, n (%)**			0.251			0.005			0.010
No formal/Primary school	3565 (76.53)	151 (80.32)		2482 (72.34)	101 (84.87)		3042 (66.80)	129 (76.33)	
Middle/High school	1006 (21.60)	34 (18.09)		875 (25.50)	16 (13.45)		1463 (32.13)	39 (23.08)	
Technical school/College or above	87 (1.87)	3 (1.60)		74 (2.16)	2 (1.68)		49 (1.08)	1 (0.59)	
**Annual household income, n (%)**			0.173			0.570			0.063
< 12,000 CNY	1679 (36.09)	79 (42.02)		1090 (31.78)	38 (31.93)		1610 (35.35)	73 (43.20)	
12,000–19,999 CNY	1151 (24.74)	43 (22.87)		691 (20.15)	27 (22.69)		1133 (24.88)	35 (20.71)	
20,000–59,999 CNY	1322 (28.42)	48 (25.53)		1162 (33.88)	30 (25.21)		1379 (30.28)	51 (30.18)	
60,000–99,999 CNY	322 (6.92)	11 (5.85)		331 (9.65)	15 (12.61)		300 (6.59)	6 (3.55)	
≥ 100,000 CNY	178 (3.83)	7 (3.72)		156 (4.55)	9 (7.56)		132 (2.90)	4 (2.37)	
**Tobacco smoking status, n (%)**			0.198			0.561			0.814
Never	3683 (79.07)	134 (71.28)		2684 (78.23)	94 (78.99)		3755 (82.45)	135 (79.88)	
Former	161 (3.46)	22 (11.70)		109 (3.18)	7 (5.88)		137 (3.01)	16 (9.47)	
Current	814 (17.48)	32 (17.02)		638 (18.60)	18 (15.13)		662 (14.54)	18 (10.65)	
Alcohol drinking weekly, n (%)	127 (2.73)	11 (5.85)	0.012	128 (3.73)	4 (3.36)	0.834	121 (2.66)	5 (2.96)	0.811
Total physical activity (METs h/d)	27.43 (15.68–39.44)	18.34 (8.40–32.51)	< 0.001	28.02 (15.00–41.67)	14.56 (5.60–31.60)	< 0.001	25.04 (14.00–38.52)	17.72 (7.49–29.57)	< 0.001
Total energy intake (kcal/week)	10.15 (8.02–13.01)	9.24 (7.32–12.33)	0.001	10.52 (7.95–13.68)	9.33 (7.65–11.52)	0.003	10.67 (8.14–13.89)	9.58 (7.36–12.73)	0.001
BMI (kg/m^2^)	23.51 (21.24–25.98)	24.05 (21.45–26.00)	0.996	24.72 (22.38–27.06)	25.42 (22.93–27.36)	0.223	23.79 (21.50–26.17)	24.81 (22.15–27.05)	0.001
SBP (mmHg)	121.67 (110.33–135.67)	129.33 (113.83–144.00)	< 0.001	123.00 (111.33–136.67)	136.17 (118.00–150.67)	< 0.001	121.33 (110.67–135.67)	135.33 (119.67–151.00)	< 0.001
DBP (mmHg)	78.67 (72.33–86.67)	81.00 (73.33–91.67)	0.007	80.00 (73.00–87.67)	83.33 (75.00–93.33)	0.002	80.33 (74.00–88.33)	86.00 (78.33–94.67)	< 0.001
FPG (mmol/L)	5.27 (4.95–5.65)	5.21 (4.90–5.57)	0.125	5.18 (4.88–5.55)	5.27 (4.90–5.74)	0.067	5.13 (4.85–5.48)	5.24 (5.00–5.64)	< 0.001
TC (mmol/L)	4.82 (4.26–5.47)	4.70 (4.05–5.46)	0.170	4.93 (4.33–5.59)	5.03 (4.44–5.98)	0.109	4.86 (4.29–5.50)	4.97 (4.41–5.65)	0.090
TG (mmol/L)	1.48 (1.06–2.16)	1.46 (1.08–2.27)	0.708	1.38 (0.99–2.05)	1.59 (1.24–1.60)	0.002	1.37 (0.99–2.00)	1.56 (1.14–2.15)	0.010
HDL-C (mmol/L)	1.47 (1.23–1.74)	1.37 (1.15–1.62)	< 0.001	1.46 (1.26–1.68)	1.43 (1.24–1.60)	0.373	1.49 (1.32–1.69)	1.50 (1.33–1.67)	0.997
LDL-C (mmol/L)	2.91 (2.35–3.46)	2.84 (2.26–3.52)	0.663	2.76 (2.26–3.37)	2.88 (2.32–3.57)	0.062	2.43 (2.02–2.91)	2.54 (2.11–3.10)	0.042
SUA (mg/dL)	5.29 (4.37–6.42)	5.79 (4.80–7.10)	< 0.001	5.33 (4.44–6.37)	5.56 (4.80–6.80)	0.008	4.95 (4.13–6.00)	5.73 (4.55–6.69)	< 0.001
Scr (µmol/L)	65.00 (56.00–76.00)	71.00 (60.00–84.50)	< 0.001	62.00 (53.00–73.00)	67.00 (58.00–77.00)	< 0.001	60.00 (53.00–70.00)	67.00 (57.00–78.00)	< 0.001
eGFR (mL/min per 1.73m^2^)	81.00 (74.00–87.00)	76.00 (69.50–82.00)	< 0.001	84.00 (78.00–90.00)	77.00 (71.00–82.00)	< 0.001	84.00 (78.00–90.00)	79.00 (73.00–85.00)	< 0.001

*BMI* body mass index; *CNY* Chinese Yuan; *CVD* cardiovascular disease; *DBP* diastolic blood pressure; *eGFR* estimated glomerular filtration rate; *FPG* fasting plasma glucose; *HDL-C* high-density lipoprotein-cholesterol; *LDL-C* low-density lipoprotein-cholesterol; *METs h/d* metabolic equivalent tasks hours/day; *SBP* systolic blood pressure; *Scr* Serum creatinine; *SUA* serum uric acid; *TC* total cholesterol; *TG* triglycerides.

Data are presented as number (percentage) for categorical variables and median (interquartile range) for continuous variables.

*P* comparing non-CVD and CVD.

**Table 2:** Basic characteristics of the Dong, Miao, and Bouyei ethnic groups by cardiovascular disease status in urban areas.

	Dong ethnic group			Miao ethnic group			Bouyei ethnic group		
Variables	non-CVD	CVD	*P*	non-CVD	CVD	*P*	non-CVD	CVD	*P*
No. of participants	1320	62		1359	77		645	36	
Age (years)	49.90 (42.77–55.92)	57.48 (54.20–63.99)	< 0.001	50.20 (42.04–56.35)	60.77 (53.97–69.42)	< 0.001	52.01 (44.69–58.09)	60.75 (55.39–71.75)	< 0.001
Men, n (%)	502 (38.03)	28 (45.16)	0.259	517 (38.04)	33 (42.86)	0.398	205 (31.78)	17 (47.22)	0.055
**Education, n (%)**			0.107			0.009			< 0.001
No formal/Primary school	194 (14.70)	13 (20.97)		234 (17.22)	21 (27.27)		93 (14.42)	15 (41.67)	
Middle/High school	521 (39.47)	26 (41.94)		612 (45.03)	36 (46.75)		302 (46.82)	14 (38.89)	
Technical school/College or above	605 (45.93)	23 (37.10)		513 (37.75)	20 (25.97)		250 (38.76)	7 (19.44)	
**Annual household income, n (%)**			0.542			0.410			0.310
< 12,000 CNY	77 (5.85)	4 (6.45)		92 (6.78)	8 (10.39)		57 (8.84)	8 (22.22)	
12,000–19,999 CNY	112 (8.51)	3 (4.84)		118 (8.70)	7 (9.09)		83 (12.87)	5 (13.89)	
20,000–59,999 CNY	337 (25.61)	14 (22.58)		422 (31.10)	24 (31.17)		196 (30.39)	5 (13.89)	
60,000–99,999 CNY	354 (26.90)	20 (32.26)		398 (29.33)	19 (24.68)		156 (24.19)	8 (22.22)	
≥ 100,000 CNY	436 (33.13)	21 (33.87)		327 (24.10)	19 (24.68)		153 (23.72)	10 (27.28)	
**Tobacco smoking status, n (%)**			0.329			0.894			0.859
Never	1078 (81.73)	46 (74.19)		1064 (78.29)	60 (77.92)		546 (84.65)	29 (80.56)	
Former	49 (3.71)	6 (9.68)		62 (4.56)	5 (6.49)		24 (3.72)	5 (13.89)	
Current	192 (14.56)	10 (16.13)		233 (17.14)	12 (15.58)		75 (11.63)	2 (5.56)	
Alcohol drinking weekly, n (%)	61 (4.62)	3 (4.84)	0.937	76 (5.59)	5 (6.49)	0.739	34 (5.27)	3 (8.33)	0.431
Total physical activity (METs h/d)	17.79 (11.20–27.83)	14.94 (8.20–23.87)	0.107	18.77 (10.88–30.15)	10.71 (5.60–17.97)	< 0.001	16.35 (9.80–27.12)	9.39 (5.85–20.11)	0.001
Total energy intake (kcal/week)	10.51 (8.68–12.85)	9.16 (7.79–11.17)	< 0.001	10.85 (8.62–13.65)	10.15 (8.65–13.39)	0.556	10.48 (8.33–13.55)	11.30 (9.92–12.95)	0.564
BMI (kg/m^2^)	24.46 (22.40–26.70)	25.13 (23.82–27.74)	0.017	24.99 (22.78–27.17)	26.09 (23.32–28.15)	0.086	24.19 (22.03–26.46)	25.82 (22.41–27.71)	0.079
SBP (mmHg)	121.00 (109.00–135.33)	130.67 (118.67–141.67)	0.001	121.67 (110.33–136.33)	131.67 (123.00–142.00)	< 0.001	122.33 (109.67–136.67)	133.33 (123.33–153.50)	< 0.001
DBP (mmHg)	80.33 (73.33–88.67)	81.00 (75.67–87.33)	0.357	81.00 (73.67–89.33)	83.33 (77.33–90.00)	0.212	81.33 (74.33–89.67)	85.17 (78.17–94.17)	0.052
FPG (mmol/L)	5.38 (5.01–5.83)	5.73 (5.33–6.86)	0.001	5.27 (4.94–5.73)	5.65 (5.23–6.68)	< 0.001	5.23 (4.93–5.75)	5.46 (5.19–6.11)	0.042
TC (mmol/L)	5.01 (4.41–5.71)	5.00 (4.42–5.70)	0.937	5.04 (4.39–5.68)	5.07 (4.40–5.97)	0.934	4.99 (4.43–5.59)	5.02 (4.42–5.89)	0.730
TG (mmol/L)	1.58 (1.15–2.44)	1.91 (1.35–2.78)	0.019	1.57 (1.11–2.29)	1.62 (1.20–2.41)	0.518	1.46 (1.04–2.08)	1.65 (1.35–2.00)	0.155
HDL-C (mmol/L)	1.43 (1.20–1.68)	1.41 (1.12–1.64)	0.260	1.45 (1.24–1.66)	1.43 (1.26–1.59)	0.424	1.50 (1.33–1.69)	1.44 (1.31–1.66)	0.473
LDL-C (mmol/L)	3.07 (2.47–3.64)	3.16 (2.41–3.73)	0.896	2.91 (2.41–3.50)	2.91 (2.39–3.75)	0.621	2.59 (2.13–3.05)	2.59 (2.22–3.25)	0.429
SUA (mg/dL)	5.66 (4.72–6.89)	6.08 (4.92–7.39)	0.039	5.56 (4.67–6.64)	5.91 (4.94–7.26)	0.010	5.51 (4.60–6.45)	5.84 (4.88–7.25)	0.136
Scr (µmol/L)	66.00 (57.00–80.00)	70.50 (59.00–89.00)	0.043	63.00 (55.00–76.00)	70.00 (59.00–85.00)	< 0.001	61.00 (53.00–72.00)	69.50 (55.00–91.00)	0.008
eGFR (mL/min per 1.73m^2^)	81.00 (75.00–88.00)	76.00 (70.00–82.00)	0.001	83.00 (77.00–89.00)	75.00 (71.00–80.00)	< 0.001	83.00 (77.00–90.00)	76.50 (71.00–82.00)	< 0.001

*BMI* body mass index; *CNY* Chinese Yuan; *CVD* cardiovascular disease; *DBP* diastolic blood pressure; *eGFR* estimated glomerular filtration rate; *FPG* fasting plasma glucose; *HDL-C* high-density lipoprotein-cholesterol; *LDL-C* low-density lipoprotein-cholesterol; *METs h/d* metabolic equivalent tasks hours/day; *SBP* systolic blood pressure; *Scr* Serum creatinine; *SUA* serum uric acid; *TC* total cholesterol; *TG* triglycerides.

Data are presented as number (percentage) for categorical variables and median (interquartile range) for continuous variables.

*P* comparing non-CVD and CVD.

### Prevalence of CVD

**Figure 1:**
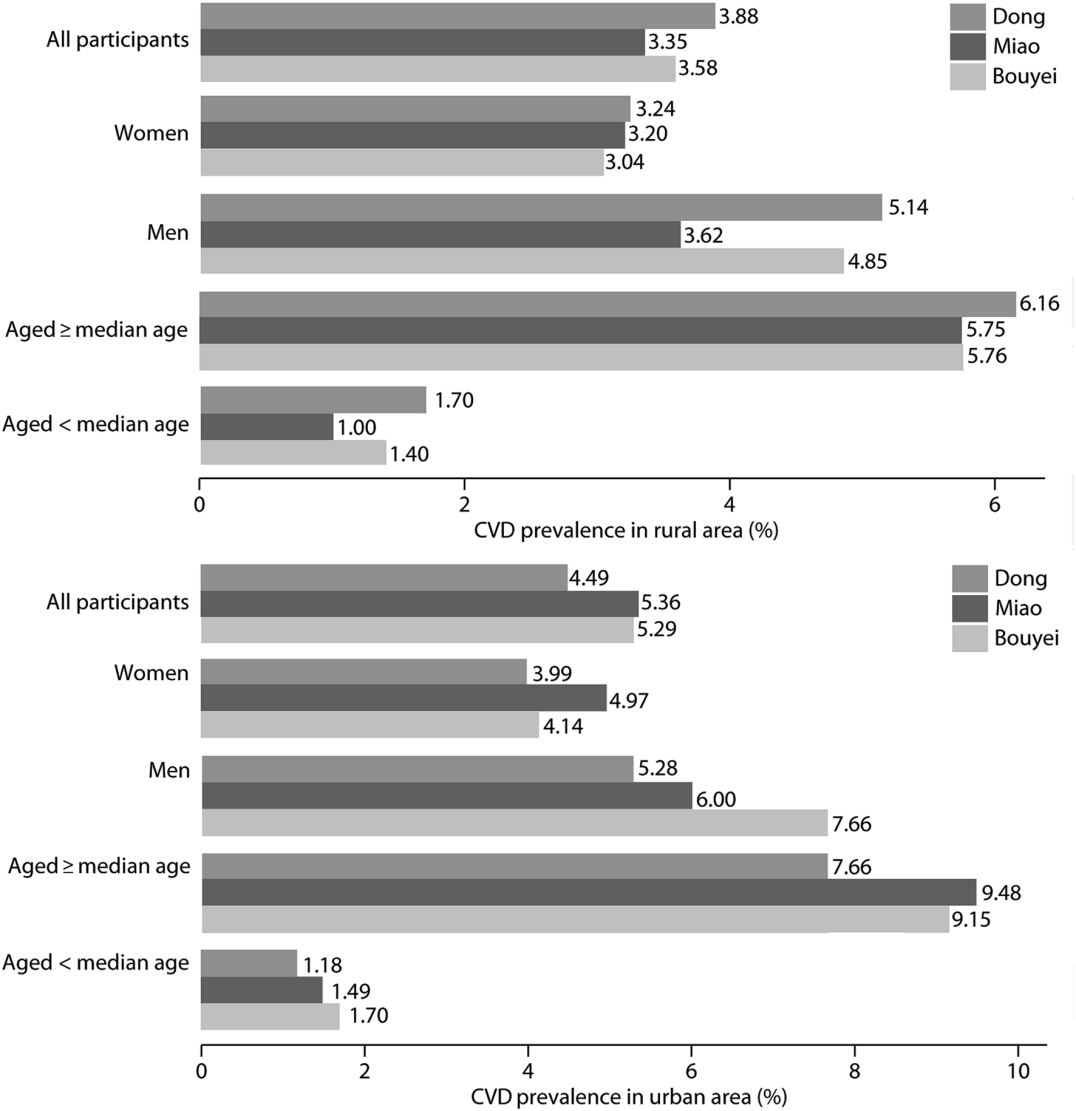
Prevalence of cardiovascular disease, coronary heart disease, and stroke in rural and urban areas. *CHD* coronary heart disease; *CVD* cardiovascular disease.

Among 16,618 people, 651 (3.92%) had CVD, which included 250 (4.01%) Dong, 196 (3.93%) Miao, and 205 (3.79%) Bouyei patients. The prevalence of CVD was 3.88%, 3.35%, and 3.58% in rural areas; in addition, 4.49%, 5.36%, and 5.29% in urban areas, among the Dong, Miao, and Bouyei ethnic groups, respectively (Fig. 1).

### Risk of CVD, CHD, and stroke in rural areas

**Figure 2:**
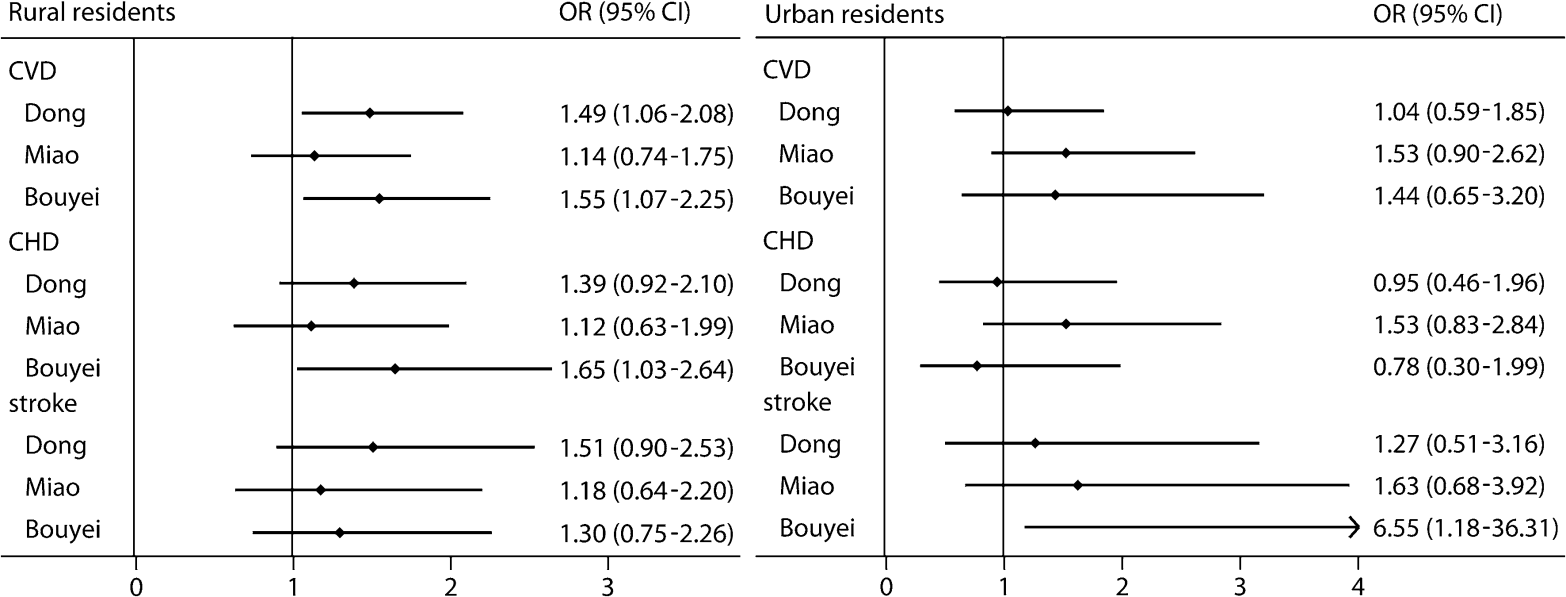
Risk of cardiovascular disease, coronary heart disease, and stroke in rural and urban areas. Adjusted for sex, age, educational attainment, annual household income, tobacco smoking status, alcohol drinking status, total physical activity, total energy intake, family history of cardiovascular disease, body mass index, and systolic blood pressure, fasting plasma glucose, and triglycerides levels. *CHD* coronary heart disease; *CI* confidence interval; *CVD* cardiovascular disease; *OR* odds ratio.

**Figure 3:**
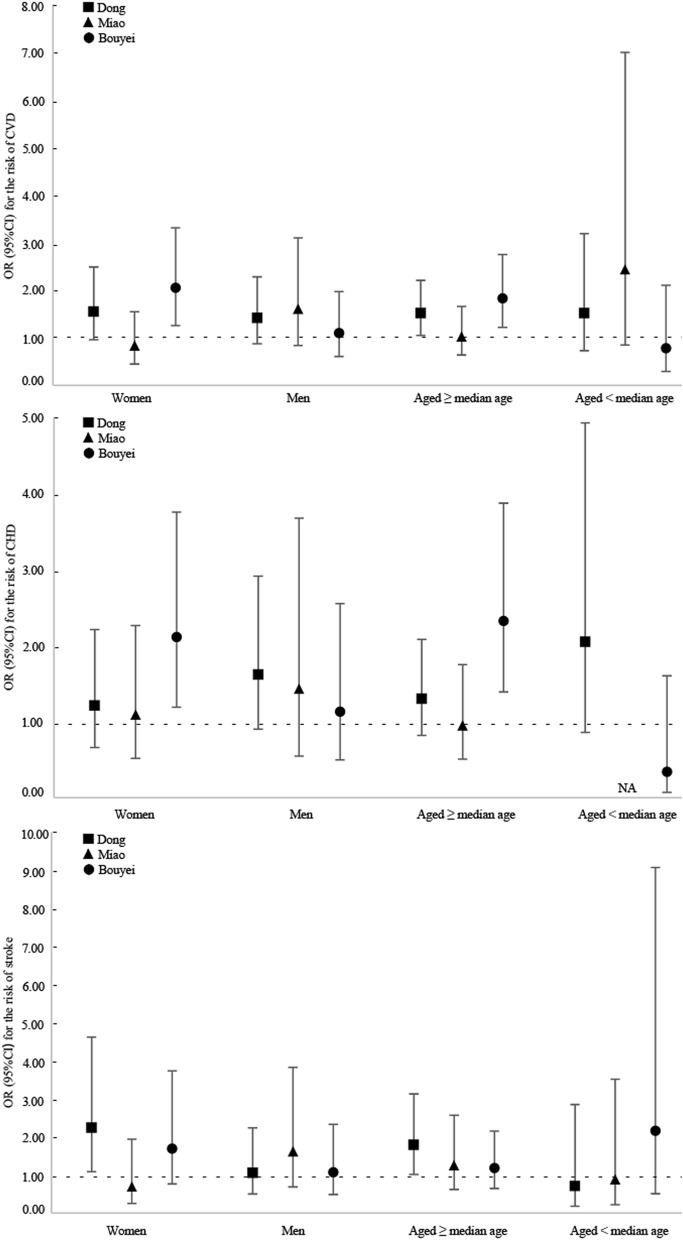
Risk of cardiovascular disease, coronary heart disease, and stroke stratified by sex and age in rural areas. Adjusted for sex, age, educational attainment, annual household income, tobacco smoking status, alcohol drinking status, total physical activity, total energy intake, family history of cardiovascular disease, body mass index, and systolic blood pressure, fasting plasma glucose, and triglycerides levels, except for the stratified variables. *CHD* coronary heart disease; *CI* confidence interval; *CVD* cardiovascular disease; *NA* not applicable; *OR* odds ratio. Median of age were 53, 51, and 51 years among the Dong, Miao, and Bouyei ethnic groups, respectively.

Figure 2 summarizes the relationship between HUA and the adjusted risk of CVD, CHD, and stroke among all three ethnic groups in rural areas. After adjusting for potential confounders, among rural residents, compared with people without HUA, an elevated risk of CVD associated with HUA was observed for the Dong (OR 1.49, 95% CI 1.06–2.08) and Bouyei (OR 1.55, 95% CI 1.07–2.25) ethnic groups (Fig. 2). Additionally, a 65% increased risk of CHD (OR 1.65, 95% CI 1.03–2.64) related to HUA was found for the Bouyei ethnic group (Fig. 2). Among the Dong and Bouyei ethnic groups, a more than two-fold risk of CVD and its subtypes associated with HUA was found in women after controlling the potential confounders (Fig. 3). In detail, among the Dong ethnic group, the present study suggested that women with HUA had a 2.24-fold increased risk of stroke (OR 2.24, 95%CI 1.09–4.62); moreover, among the Bouyei ethnic group, we observed a 2.05-fold elevated risk of CVD (OR 2.05, 95%CI 1.26–3.31) and 2.11-fold increased risk of CHD (OR 2.11, 95%CI 1.19–3.75) in women with HUA than people without HUA. Additionally, in older people, a 52% increased risk of CVD (OR 1.52, 95%CI 1.05–2.21) and a 79% elevated risk of stroke (OR 1.79, 95%CI 1.02–3.13) was detected in HUA patients among the Dong ethnic group; furthermore, those with HUA among the Bouyei ethnic group had a 83% increased risk of CVD and a 132% elevated risk of CHD with the multi-adjusted OR and 95%CI of 1.83 (1.22–2.75) and 2.32 (1.39–3.87), respectively. However, the relationship between HUA and the risk of CVD, CHD, and stroke was not statistically significant in men and middle-aged participants, as well as in the Miao ethnic group (Fig. 3).

### Risk of CVD, CHD, and stroke in urban areas

**Figure 4:**
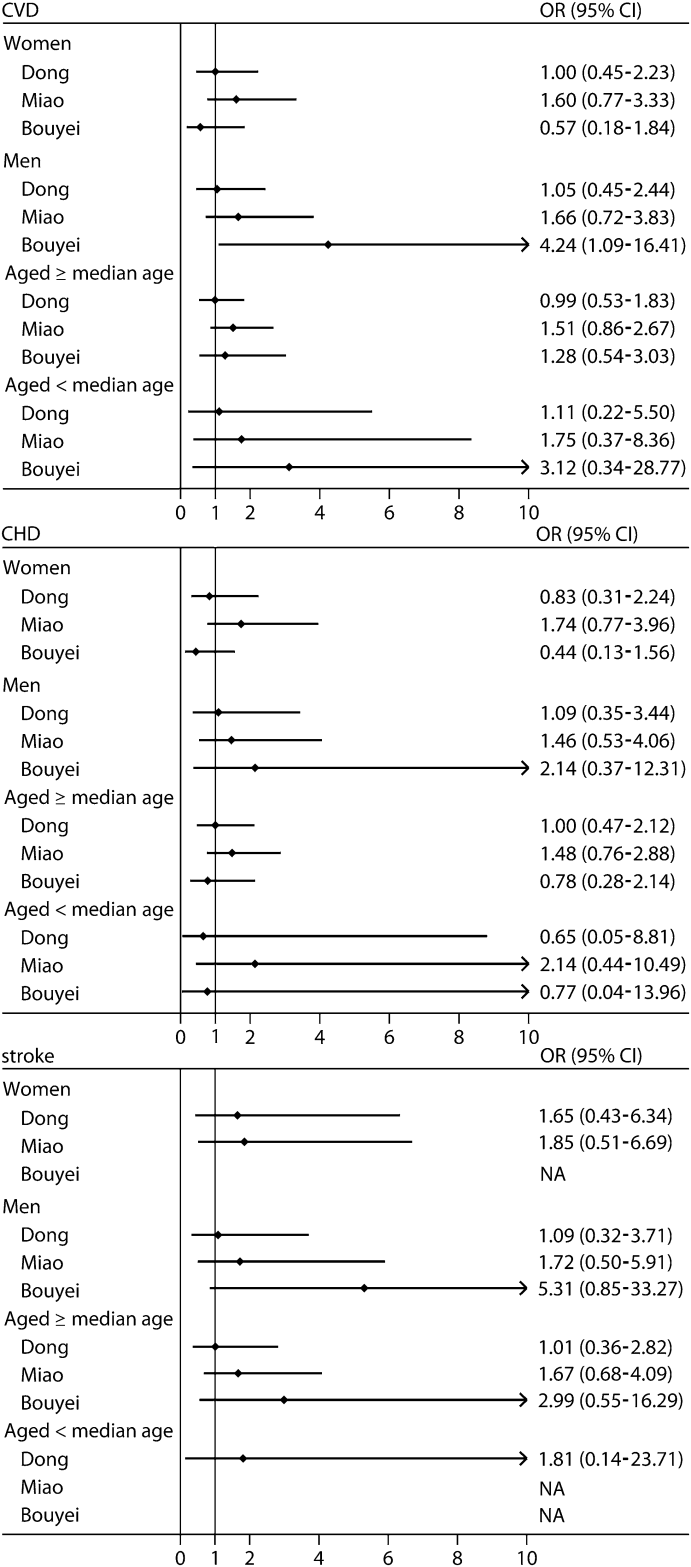
Risk of cardiovascular disease, coronary heart disease, and stroke stratified by sex and age in urban areas. Adjusted for sex, age, educational attainment, annual household income, tobacco smoking status, alcohol drinking status, total physical activity, total energy intake, family history of cardiovascular disease, body mass index, and systolic blood pressure, fasting plasma glucose, and triglycerides levels, except for the stratified variables. *CHD* coronary heart disease; *CI* confidence interval; *CVD* cardiovascular disease; *NA*, not applicable; *OR* odds ratio. Median of age were 50, 51, and 53 years among the Dong, Miao, and Bouyei ethnic groups, respectively.

No other significant association between HUA and the risk of was CVD, CHD, and stroke in urban areas was found, except for a 6.55-fold increased risk of stroke (OR 6.55, 95% CI 1.18–36.31) in total urban residents with HUA (Fig. 2) and a 4.24-fold increased risk of CVD (OR 4.24, 95%CI 1.09–16.41) in men with HUA (Fig. 4) among the Bouyei ethnic group as compared with people without HUA, after adjusting for some possible confounding factors.

## Discussion

This study included 16,618 people without a reduced estimated glomerular filtration rate and revealed rural–urban disparities in the HUA-CVD relationship in Southwest China. Our study suggested a novel perspective that HUA was associated with a higher risk for CVD in Chinese Bouyei and Dong adults. In detail, among the Bouyei ethnic group, the present data suggested that HUA was positively associated with an increased risk of CVD and CHD in rural women and rural older people. Among the Dong ethnic group, an increased risk of CVD and stroke related to HUA in the rural elderly and an elevated risk of stroke associated with HUA in rural women were observed. Among the Miao ethnic group, there was no evidence of the relationship.

Conflicting reports (positive relationship or no statistical significance) of epidemiological surveys existed in the association between HUA and the risk of CVD^3,4,8,13,14,24^. Few observational studies have examined the potential rural–urban differences of the association between HUA and the risk of CVD and conflicting reports existed^8,9,25^; additionally, sex-specific and age-specific differences in the relationship between residences warrant further exploration^8–10,13^. A positive association between HUA and 10-year CVD risk was observed in rural women, rural men, and urban women but not urban men aged 40–70 years in African populations^8^. In addition, a cross-sectional epidemiological survey in 11,731 rural Han Chinese adults aged 35 years and older demonstrated a positive correlation between HUA and an increased risk of stroke in women but not men^9^. Whether the association varies by ethnicity, especially in minority areas in China, is unclear. In the present study, a positive association of HUA with CVD risk in rural people, particularly in women and older adults living in rural areas, was observed among the Dong ethnic group, similar to that observed among the Bouyei ethnic group. However, we did not find a positive association among the Miao ethnic group. Racial/ethnic differences in lifestyle, intrinsic biologic differences, disparities in risk factor management, or gene expression could affect the association^26,27^.

Rural areas are relatively short of medical and health services compared with urban residences^28^. There is better health policy and service capacity in the prevention and control of CVD distributed in eastern and southern areas in China^29^. The inadequate use of anti-HUA treatment or CVD medications in rural residents is evident, especially in southwest ethnic Chinese groups^28,29^. Available essential SUA-lowering drugs or CVD medicines in primary health care institutions and medical help of general practitioners timely and fully could be associated with a reduction or prevention of the CVD risk related to HUA. Additionally, women are likely to exhibit an excess burden of CVD associated with HUA compared with men based on present findings. The sex difference in the association may be related to the inherent biological differences. Sex-specific changes in the sex hormone visceral fat and muscle mass, which regulate SUA metabolism, were observed and affected the relationship. Health physical activity or scientific guidance of professionals is lacking, and professionals are less aware of turning to lifestyle intervention, which is more evident in Guizhou Province, particularly in elderly people^29^. Renal impairment, extensive vascular damage, endothelial dysfunction, reduced nitric oxide availability, or vascular inflammation promotion^30–33^ associated with HUA, could result in atherosclerosis. These phenomena are more evident in older participants than in young and middle-aged people. The discrepancy in the use of high purine foods may contribute to the differences in the relationship. The degree of emphasis given to the above aspects may influence ethnic differences in the relationship. In addition, the gene expression of the three ethnic groups included in this study may also be different. A genome-wide association study based on the project is now in progress, and we will further explore the possible impact of genes on the association of HUA with CVD risk.

As mentioned previously, there is a lack of observational studies comprehensively evaluating the relation of HUA with CVD in rural and urban areas, and the present findings have important clinical and public health implications. From medical practice point of view, this study clearly indicates that screening for HUA in rural residents, especially in women older adults living in rural areas, among Dong and Bouyei ethnicities, the major part of the high-risk rural people for developing CVD, could be recognized and cured. Our study provides valuable information in clinical treatment that early-onset HUA patients need more stringent anti-HUA therapy as early as possible to reduce the burden of CVD and loss of life. There are readily effective and available approaches to mitigate the risk of long-term SUA management or, notably, early anti-HUA treatment for this population. Available essential SUA-lowering drugs or CVD medicines in primary health care institutions and the timely and full medical help of general practitioners could be associated with reducing or preventing the CVD risk related to HUA. Self-awareness of the condition is the first step to modifying behaviour and lifestyle changes, and more intensive lifestyle interventions should also be actively encouraged. Moreover, there is a need to bridge the disconnect between policies that address HUA treatment among Han adults while overlooking the remarkable cardiovascular risks of HUA in ethnic Chinese groups.

There are several strengths to the current study. All participants were from an ethnic Chinese area with distinctive ethnic characteristics and approximately equal proportions of Bouyei, Dong, and Miao adults, making it suited to examining ethnic differences in the risk of CVD related to HUA. Insufficient evidence from potential rural–urban disparities in the HUA-CVD relationship existed in previous studies.

The present study has several limitations; the analyses showed a relevant correlation for the risk of CVD associated with HUA. It is difficult to analyse a cause-effect relationship because of the present study design. Additionally, data regarding subtypes of CHD and stroke were not available; therefore, we could not investigate whether the effect of HUA on the risk of CVD differed according to subtypes of CHD and stroke. The lack of repeated measurements of the levels of SUA and Scr may produce intraindividual variability. Additionally, there was no available information about ambulatory HUA monitoring data, and further evidence is required to establish the association between HUA and the long-term risk of CVD. In addition, residual or unmeasured confounding may be present after adjustment for covariates.

## Conclusions

In conclusion, rural residents among the Dong and Bouyei ethnic groups, had an increased risk of CVD associated with HUA, this is particularly evident in women and elderly individuals living in a rural area. Our data suggest a need to better target cardiovascular risk among those with early-onset HUA. Both primordial prevention and therapeutic management of HUA are important in reducing CVD risk. In the coming decades, HUA patients living in minority areas may tend to overlook potential health hazards, which translates into a high disease burden of CVD, particularly in Southwest China. Ethnicity, residence, sex-, and age-specific strategies could be developed to reduce the risk of CVD related to HUA. Controlling the level of SUA within the normal range may be the key to HUA treatment. If further elaborated in future research, the hypothesis emerging from this study could have far-reaching consequences on planning CVD prevention, which is important in ethnic Chinese groups.

## Data Availability

The datasets used and/or analyzed during the current study are available from the corresponding author on reasonable request.

## Abbreviations

CHD: Coronary heart disease
CI: Confidence interval
CVD: Cardiovascular disease
eGFR: Estimated glomerular filtration rate
HUA: Hyperuricaemia
OR: Odds ratio
Scr: Serum creatinine
SUA: Serum uric acid
